# Potential effects of UV radiation on photosynthetic structures of the bloom-forming cyanobacterium *Cylindrospermopsis raciborskii* CYRF-01

**DOI:** 10.3389/fmicb.2015.01202

**Published:** 2015-10-30

**Authors:** Natália P. Noyma, Thiago P. Silva, Hélio Chiarini-Garcia, André M. Amado, Fábio Roland, Rossana C. N. Melo

**Affiliations:** ^1^Laboratory of Aquatic Ecology, Department of Biology, Federal University of Juiz de ForaJuiz de Fora, Brazil; ^2^Laboratory of Cellular Biology, Department of Biology, Federal University of Juiz de ForaJuiz de Fora, Brazil; ^3^Laboratory of Structural Biology and Reproduction, Department of Morphology, Federal University of Minas GeraisBelo Horizonte, Brazil; ^4^Laboratory of Limnology, Department of Oceanography and Limnology, Federal University of Rio Grande do NorteNatal, Brazil

**Keywords:** ultraviolet radiation, cyanobacteria, cell death, thylakoid membranes, transmission electron microscopy, cell viability

## Abstract

Cyanobacteria are aquatic photosynthetic microorganisms. While of enormous ecological importance, they have also been linked to human and animal illnesses around the world as a consequence of toxin production by some species. *Cylindrospermopsis raciborskii*, a filamentous nitrogen-fixing cyanobacterium, has attracted considerable attention due to its potential toxicity and ecophysiological adaptability. We investigated whether *C. raciborskii* could be affected by ultraviolet (UV) radiation. Non-axenic cultures of *C. raciborskii* were exposed to three UV treatments (UVA, UVB, or UVA + UVB) over a 6 h period, during which cell concentration, viability and ultrastructure were analyzed. UVA and UVA + UVB treatments showed significant negative effects on cell concentration (decreases of 56.4 and 64.3%, respectively). This decrease was directly associated with cell death as revealed by a cell viability fluorescent probe. Over 90% of UVA + UVB- and UVA-treated cells died. UVB did not alter cell concentration, but reduced cell viability in almost 50% of organisms. Transmission electron microscopy (TEM) revealed a drastic loss of thylakoids, membranes in which cyanobacteria photosystems are localized, after all treatments. Moreover, other photosynthetic- and metabolic-related structures, such as accessory pigments and polyphosphate granules, were damaged. Quantitative TEM analyses revealed a 95.8% reduction in cell area occupied by thylakoids after UVA treatment, and reduction of 77.6 and 81.3% after UVB and UVA + UVB treatments, respectively. Results demonstrated clear alterations in viability and photosynthetic structures of *C. raciborskii* induced by various UV radiation fractions. This study facilitates our understanding of the subcellular organization of this cyanobacterium species, identifies specific intracellular targets of UVA and UVB radiation and reinforces the importance of UV radiation as an environmental stressor.

## Introduction

Cyanobacteria are a widely distributed group of aquatic photosynthetic organisms. Over 2.4 billion years ago, oxygenic photosynthesis carried out by primitive cyanobacteria transformed early Earth’s reducing atmosphere into an oxidizing one (Rasmussen et al., 2008). Their widespread ecological importance includes symbiotic interactions (Adams, 2002) and impacts on nutrient cycling (e.g., fixing atmospheric nitrogen) (Capone et al., 1999).

Cyanobacteria species frequently form massive, harmful blooms, which contribute to a reduction in water quality as dissolved oxygen in the water is depleted. In turn, secondary problems arise such as fish mortality and the discharge of toxic substances (Carmichael, 2001). As a consequence, the presence of toxic cyanobacterial blooms in natural waters used for drinking or recreational purposes represents a serious risk to human health (Funari and Testai, 2008; Zanchett and Oliveira-Filho, 2013; Gehringer and Wannicke, 2014).

The bloom-forming cyanobacterium, *C. raciborskii*, has attracted considerable attention due to its broad distribution and recent geographic expansion, as well as its ability to produce potent toxins such as hepatotoxins and neurotoxins (Molica et al., 2002; Carneiro et al., 2013). Although it is considered a tropical/subtropical species, the prevalence of *C. raciborskii* in temperate climatic zones has rapidly increased over the past two decades (Hamilton et al., 2005; Figueredo and Giani, 2009; Everson et al., 2011; Sinha et al., 2012). Overall, the geographical expansion of *C. raciborskii* has been partially attributed to a number of factors, which include increasing temperatures and eutrophication (reviewed in Sinha et al., 2012). While the effects of increasing temperatures and other aspects, such as nutrient availability, on *C. raciborskii* have been extensively investigated (reviewed in Sinha et al., 2012), the effect of ultraviolet (UV) radiation, which is an important source of ionization energy in the biosphere, on this species has not.

Although very small proportions of solar UV radiation contribute to the total irradiance of the Earth’s surface (UVC: 0%, 100–290 nm; UVB: <1%, 280–315 nm; and UVA: <7%, 315–400 nm), this portion of the solar spectrum is highly energetic (Kirk, 1994). Anthropogenic activities over the last three decades have contributed to the depletion of the ozone layer and, therefore, the consequent increase in solar UV radiation reaching the Earth’s surface has become an important issue (Crutzen, 1992; Madronich, 1992; Kerr and McElroy, 1993). Despite international efforts made to reverse such negative processes, levels have not returned to those seen prior to the 1980s; the timing of a return to pre-1980 UV levels cannot be precisely predicted (McKenzie et al., 2011).

Several processes are affected by UV irradiance. For instance, UVB induces a reduction in the metabolism of heterotrophic bacteria (e.g., Amado et al., 2015), as well as aquatic primary producers, such as cyanobacteria, mainly due to DNA damage (Buma et al., 2001; Helbling et al., 2001; Rastogi et al., 2010). Furthermore, detrimental effects by UVA on phytoplankton have been observed on primary production, pigment degradation and changes in nitrogen metabolism (Kim and Watanabe, 1994; Döhler and Buchmann, 1995; Palffy and Voros, 2006).

In the present work, we investigated the potential effects of different wavelength bands of UV radiation on the concentration, viability and ultrastructure of a *C. raciborskii* strain. Our data demonstrate that UV radiation, mainly UVA, induces drastic damage to the cyanobacterium’s cytoplasmic thylakoid membranes and their associated pigments, leading to cell death.

## Materials and Methods

### Cyanobacteria Strain and Culture

The cyanobacterium *C. raciborskii* (CYRF-01) was obtained from the culture collection of the *Laboratório de Ecofisiologia e Toxicologia de Cianobactérias (IBCCF –UFRJ*, Brazil). This *C. raciborskii* strain is able to produce saxitoxins and gonyautoxins (Ferrão-Filho et al., 2009). A non-axenic cyanobacterial stock culture was maintained in sterile ASM-1 growth medium in 300-mL Erlenmeyer flasks placed in a climate-controlled room at 25°C, 35 μmol photons m-2 s^-1^, with a light–dark cycle of 12:12 h (Gorham et al., 1964).

### UV Irradiation Exposure

In order to evaluate the effects of different UV wavebands, we administered the following treatments: UVA + UVB (280–400 nm), UVA (315–400 nm), UVB (280–315 nm) and control (Photosynthetically Active Radiation [PAR]; 400–700 nm). For each group, samples of *C. raciborskii* from the same stock culture in exponential growth phase were re-suspended in 40 mL of fresh ASM-1 medium (Gorham et al., 1964) at an initial concentration of 10^6^ cells/mL. Subsequently, groups were exposed to artificial UV radiation supplied by UVA (TL 40/05; Philips; emission peak at 365 nm) and UVB (TL 20/01; Philips; emission peak at 312 nm) lamps. The UV intensities used in experiments were 11.8 Wm^-2^ (UVA) and 0.54 Wm^-2^ (UVB). These values were based on natural solar radiation measurements taken during May, 2009 in Juiz de Fora City (21°45′51″ S) in southeast Brazil. UVA + UVB and UVB treatments were performed in borosilicate glass Erlenmeyers (40 mL) incubated under UVA and/or UVB lamps. UVA treatment was performed in quartz flasks (40 mL) incubated under UVA lamps, while the control treatment was performed in quartz flasks (40 mL) under PAR radiation using the same conditions as the stock culture. According to our measurements, borosilicate glass decreased UVB intensity by 50% and UVA by 10% while quartz glass has a transmittance of ∼90% for UVA, UVB, and PAR radiations (Six et al., 2007). The distance between the UV lamps and flasks (∼30 cm) was calculated using a radiometer (IL 1400A; International Light Technologies, Peabody, MA, USA) in order to ensure the radiation intensity used was in accordance with previous descriptions. Treatments were performed during 6 h at room temperature (RT; 20 ± 1°C) and all samples were carefully homogenized prior to analysis. All experiments were performed in triplicate.

### Cell Concentration

Cyanobacteria samples were taken from each experimental group for cell concentration evaluations as previously described (Sipaúba-Tavares and Rocha, 2003). Briefly, samples were fixed with Lugol solution and cells counted in a light microscope (BX 41; Olympus, Tokyo, Japan) at 400 × magnification using an improved Neubauer hemocytometer (Sipaúba-Tavares and Rocha, 2003). Analyses were performed at different time points of UV exposure (0, 2, 4, and 6 h).

### Cell Viability

Cell membrane integrity was investigated using a fluorescent probe (*Baclight*) as an indicator of cell viability (Boulos et al., 1999). Samples were collected from each group before UV exposure (0 h) and at the end of 6 h. The percentage of live/viable and dead/non-viable cells was determined using a LIVE/DEAD BacLight Viability kit (Molecular Probes, Inc, ThermoFisher Scientific, Eugene, OR, USA), which contains a mixture of fluorescent dyes, SYTO^^®^^ 9 and propidium iodide. These probes differ both in their spectral characteristics and in their ability to penetrate healthy bacterial cell membranes. Cells with intact membranes (live cells) stain green and those with damaged membranes (dead cells) stain red (Barbesti et al., 2000; Joux and Lebaron, 2000; Hoefel et al., 2003; Berney et al., 2007). A mixture of equal volumes of each stain (total volume of 0.9 μL) was added to 300 μL of each sample and incubated for 20 min in the dark. Slides (*n* = 3) for each time point were prepared in a cytocentrifuge (Shandon Cytospin 4, Thermo Electron Corporation, Madison, WI, USA), as previously described (Silva et al., 2014), at 28 × g for 5 min at medium acceleration and then evaluated under a fluorescence microscope (BX-60, Olympus, Tokyo, Japan) at 450–480 nm excitation wavelengths, which enable simultaneous fluorescence visualization of live and dead cells. This fluorescence did not cross talk with chlorophyll autofluorescence emitted by cyanobacteria using green (510–550 nm excitation wavelengths) and UV (330–385 nm excitation wavelengths) filters (Supplementary Figure S1). For each group, 30 cyanobacterial filaments were counted and the percentage of live/dead cells determined. Images were taken using an Evolution VF (Media Cybernetics, Rockville, MD, USA) digital camera and Image Pro-Plus 5.0 software (Media Cybernetics).

### Transmission Electron Microscopy (TEM)

Samples collected for each group at the 6 h time point were immediately fixed in a mixture of freshly prepared aldehydes (1% paraformaldehyde and 1% glutaraldehyde) in 0.1 M phosphate buffer, pH 7.3, for 1 h at RT, washed twice in the same buffer at 1500 × g for 10 min, and stored at 4°C for subsequent use. After fixation, agar embedding was performed as previously described (Barros et al., 2010; Silva et al., 2014), so that uniformly distributed specimens could be processed as easily handled blocks of cells. Briefly, samples were centrifuged at 1,500 ×*g* for 1 min. They were then re-suspended in molten 2% agar (Merck, Darmstad, Germany) for further processing. Agar pellets containing water specimens were post-fixed in a mixture of 1% phosphate-buffered osmium tetroxide and 1.5% potassium ferrocyanide (final concentration) for 1 h prior to dehydration in graded ethanols (70, 95, and 100%), and infiltration and embedding in a propylene oxide-Epon sequence (PolyBed 812, Polysciences, Warrington, PA, USA) (Melo et al., 2006). After polymerization at 60°C for 16 h, thin sections were cut using a diamond knife on an LKB ultramicrotome (LKB Instruments, Gaithersburg, MD, USA). Cyanobacteria were examined using a transmission electron microscope (Tecnai Spirit G12; FEI Company, Eindhoven, The Netherlands) at 80 kV.

### Quantitative Ultrastructural Studies

To study the ultrastructural alterations potentially induced by UV radiation exposure, a total of 64 electron micrographs were randomly taken at magnifications of 9,300× to 30,000×. The following data were quantitated: (i) total cytoplasmic area; (ii) cytoplasmic area occupied by thylakoids; (iii) total number and area of polyphosphate granules; and (iv) total number of polyhedral bodies (carboxysomes). Quantitative analyses were performed using software Image J 1.41 (National Institutes of Health, Bethesda, MD, USA).

### Statistical Analysis

Cell concentration, the percentage of viable and non-viable cells and ultrastructural morphometric data were compared by one-way ANOVA, followed by Tukey’s comparison test using Prism 6.01 software (GraphPad software, San Diego, CA, USA).

## Results

### UVA and UVA + UVB Affect Cell Concentration

**FIGURE 1:**
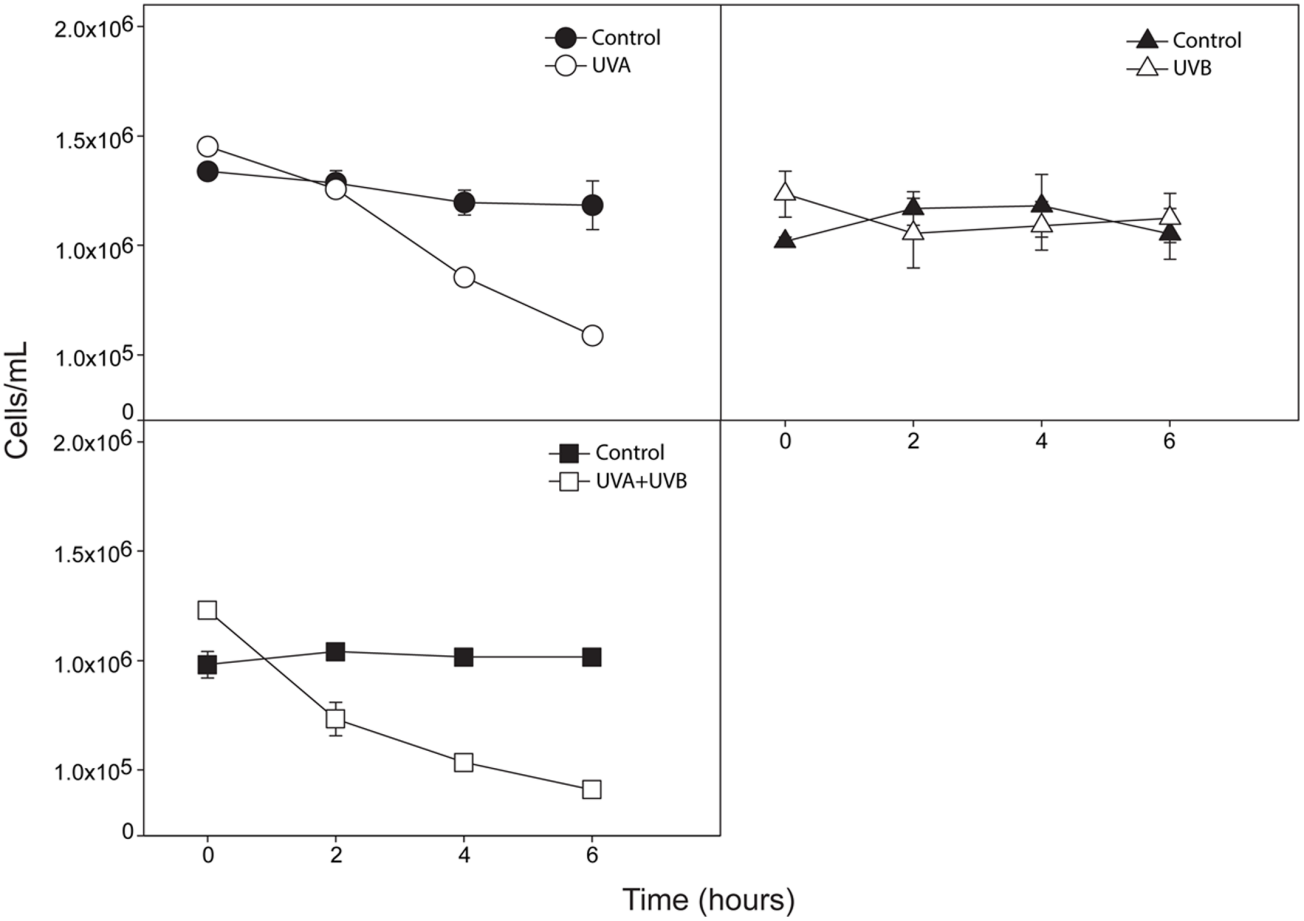
**Cell concentration evaluation after 6 h of exposure to ultraviolet radiation (UVA, UVB, and UVA + UVB).** For each time point, the concentrations of UV-treated cultures (UVA, *n* = 3; UVB, *n* = 3 and UVA + UVB, *n* = 3) and their respective controls (total *n* = 9) were analyzed after fixation and cells counted by light microscopy using a hemocytometer. All cultures were in the exponential phase of growth. Data are expressed as mean ± SD (*n* = 3). Open symbols represent treatments whereas filled symbols represent control groups.

Both UVA and UVA + UVB treatments led to significant decreases in *C. raciborskii* cell concentrations of 56.44 and 64.39%, respectively, after 6 h of treatment compared to controls (*P* < 0.001; **Figure 1**). In contrast, UVB treatment did not induce any significant change in cell concentration (*P* = 0.4894; **Figure 1**).

### UV Radiation Induces *C. raciborskii* Death

**FIGURE 2:**
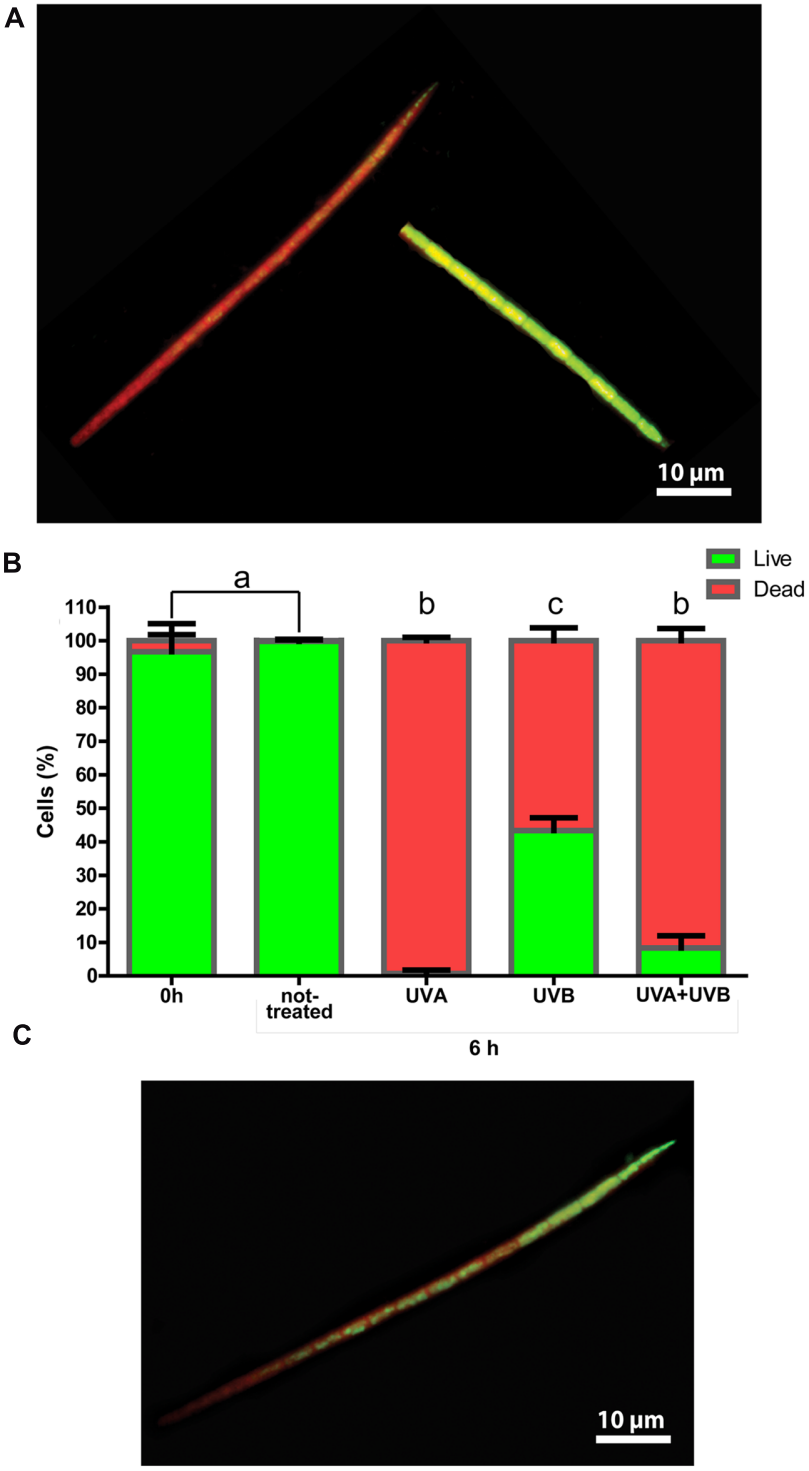
**Cell viability of *Cylindrospermopsis raciborskii* exposed to UV radiation.** In **(A)**, live (green) and dead (red) cyanobacteria are observed. **(B)** After 6 h, all treatments induced significant cell death compared to untreated and 0 h groups (*P* < 0.0001 for all). Letters indicate a significant difference (*P* < 0.0001). In **(C)**, both live and dead cells are seen on the same filament (after UVB treatment). Samples were stained with Syto 9 and propidium iodide (BacLight). Live and dead cells were viewed simultaneously, and 30 filaments/group were counted by fluorescence microscopy.

To evaluate if the observed decreases in cell concentrations at 6 h were due to cell death, we next investigated cell viability (at 0 and 6 h) using a marker for plasma membrane integrity (Decamp and Rajendran, 1998). Live and dead cyanobacteria were observed in both untreated and treated groups (**Figures 2A,B**). However, all UV treatments induced a significant increase in the percentage of dead cells after 6 h of exposure (**Figure 2B**) compared to the control group (*P* < 0.0001). Radiation induced cell death in 91.65, 99.28, and 56.63% of cells for UVA + UVB, UVA, and UVB treatments, respectively. We also evaluated the viability of each single cell in each filament. Viable and non-viable cells were observed within the same cyanobacterial filament (**Figure 2C**).

### Ultrastructure of Untreated *C. raciborskii*

**FIGURE 3:**
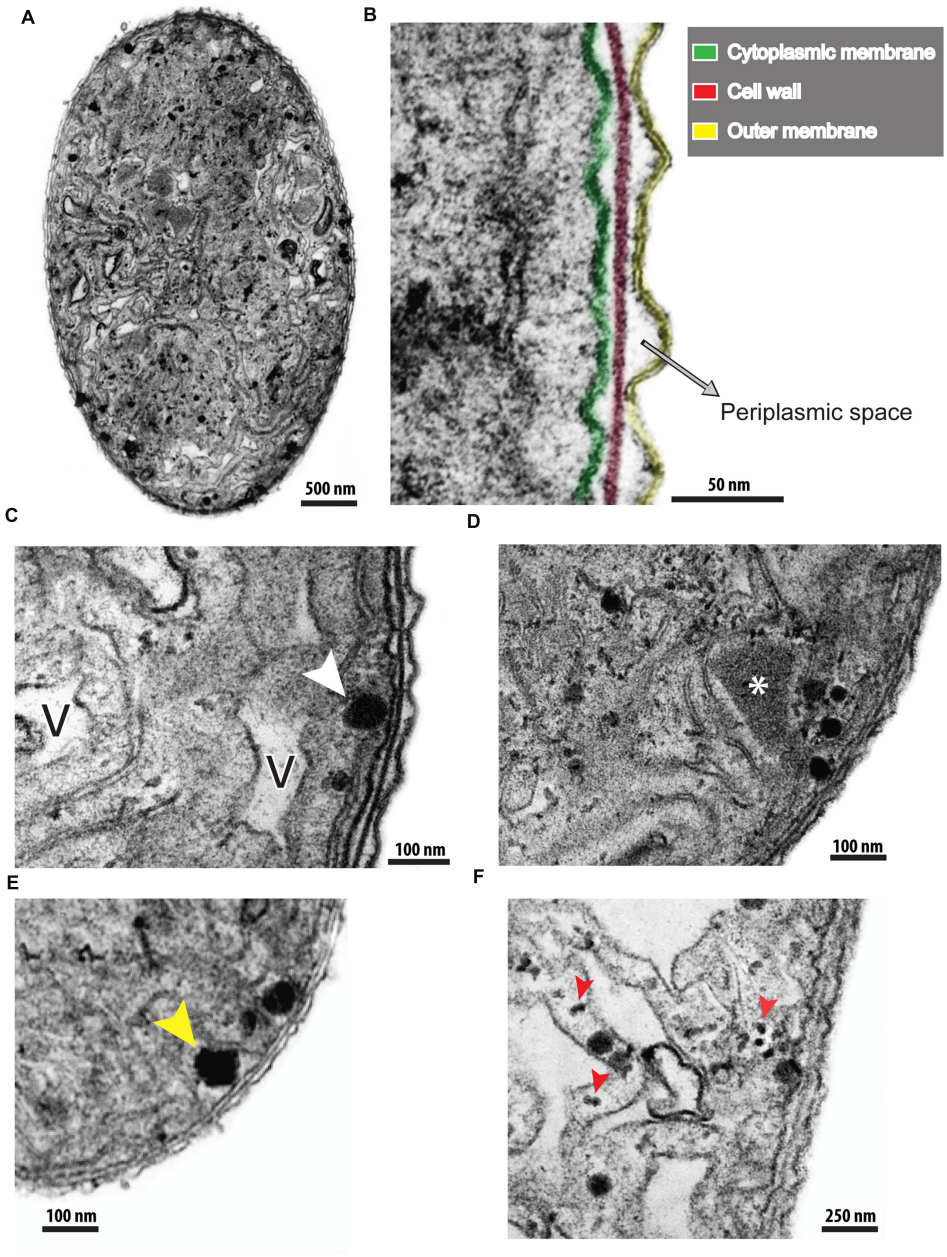
**Different views of untreated *C. raciborskii* cells observed by transmission electron microscopy (TEM).** In **(A)**, a cross-section of a cell shows a general view of the cytoplasm, cell envelope and their organization. **(B)** At higher magnification, the structure of the cellular envelope is more clearly revealed. Note the cytoplasmic membrane (green), cell wall (red) and outer membrane (yellow). **(C–E)** Cytoplasmic structures such as air vesicles (V), lipid bodies (white arrowhead), polyhedral bodies (^∗^), and polyphosphate granules (yellow arrowhead) are observed. In **(F)**, the thylakoid membrane structure is seen at high magnification. Phycobilisomes (red arrowheads) are viewed as small electron-dense dots in association with thylakoid membranes.

**FIGURE 4:**
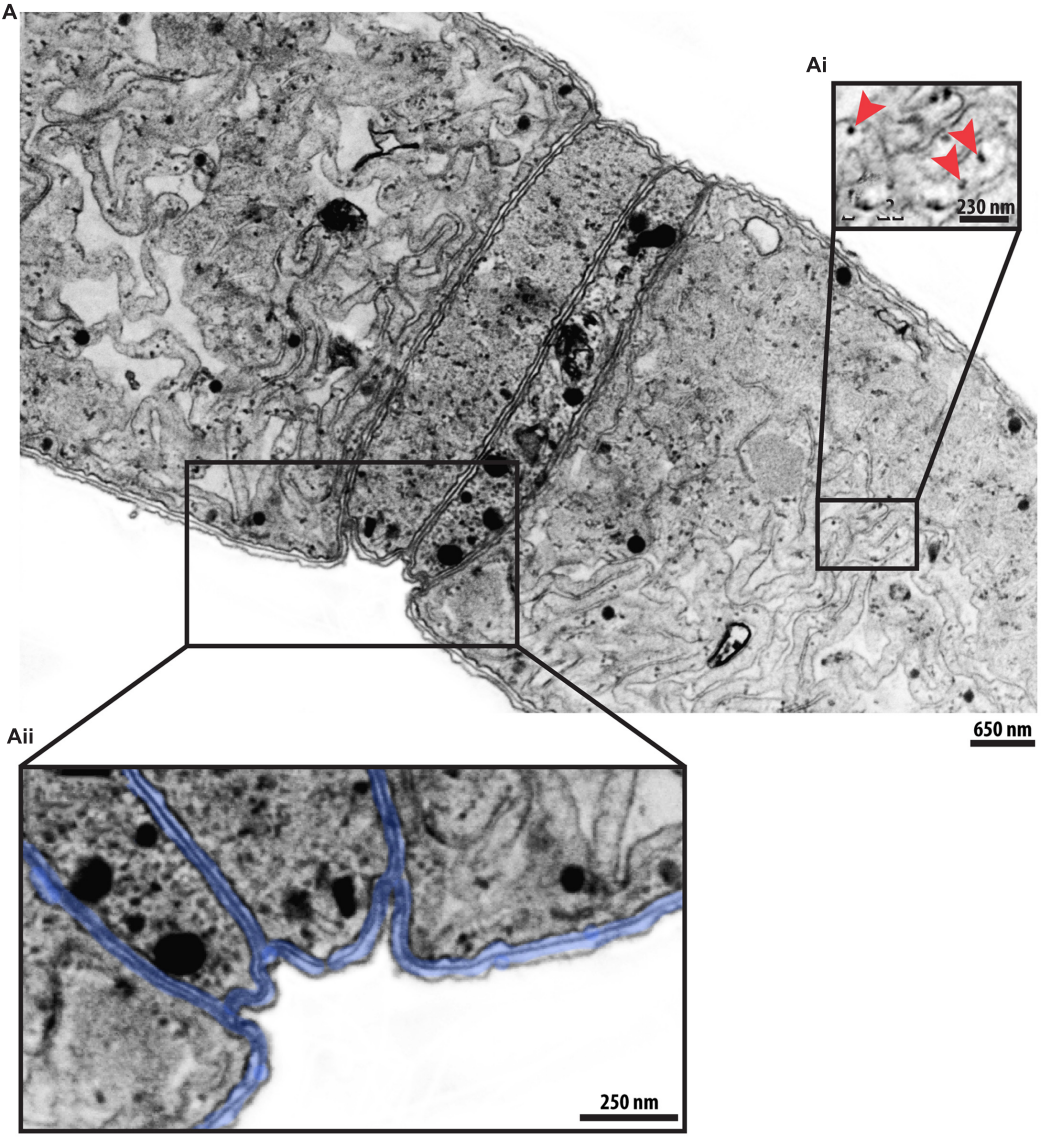
**A representative *C. raciborskii* filament seen by transmission electron microscopy (TEM). (A,Aii)** A longitudinal section shows several cells tightly associated with each other by their envelopes (highlighted in blue in **Aii**). Thylakoid membranes with phycobilisomes (indicated by arrowheads in **Ai**) are clearly visible.

Because this work was, to our knowledge, the first to investigate in extensive detail the ultrastructure of *C. raciborskii*, untreated organisms (controls) were firstly carefully analyzed (**Figures 3A–E**). Similar to other filamentous cyanobacteria, this species was characterized by an envelope composed of three layers: an inner membrane (plasma membrane), an intermediate (cell wall) and an outer membrane with a mucilaginous sheath on its extracellular surface (**Figure 3B**). Longitudinal and oblique sections clearly showed filament organization, with cells closely interrelated by this envelope (**Figures 4A,Aii**).

The cytoplasm of *C. raciborskii* was characterized by a large number of thylakoid membranes and thylakoid-associated round structures (phycobilisomes), the sites of accessory photosynthetic pigments (phycocyanin and/or phycoerythrin; **Figures 3F** and **4A,Ai**). Other structures frequently identified in the cytoplasm were: gas vesicles (**Figure 3C**), lipid bodies (**Figure 3C**), polyhedral bodies, also termed carboxysomes (**Figure 3D**) and polyphosphate granules (**Figure 3E**). Gas vesicles, structures involved with body buoyancy, appeared as vacuoles of different sizes and shapes, and contained an electron-lucent content (**Figure 3C**). Polyhedral bodies, structures involved in the process of carbon fixation during photosynthesis (reviewed in Cannon et al., 2001), were observed isolated, or in groups, in association with thylakoid membranes (**Figures 3D**). TEM quantitative analysis revealed 3.41 ± 1.06 carboxysomes per cell section (mean ± SEM, *n* = 20 cell sections). Polyphosphate granules were seen as roughly rounded structures, characterized by aggregates of electron-dense phosphates, with an irregular surface and a diameter of about 0.3 μm (**Figure 3E**).

### Cyanobacteria Photosynthetic Structures are Drastically Damaged by UV Radiation

**FIGURE 5:**
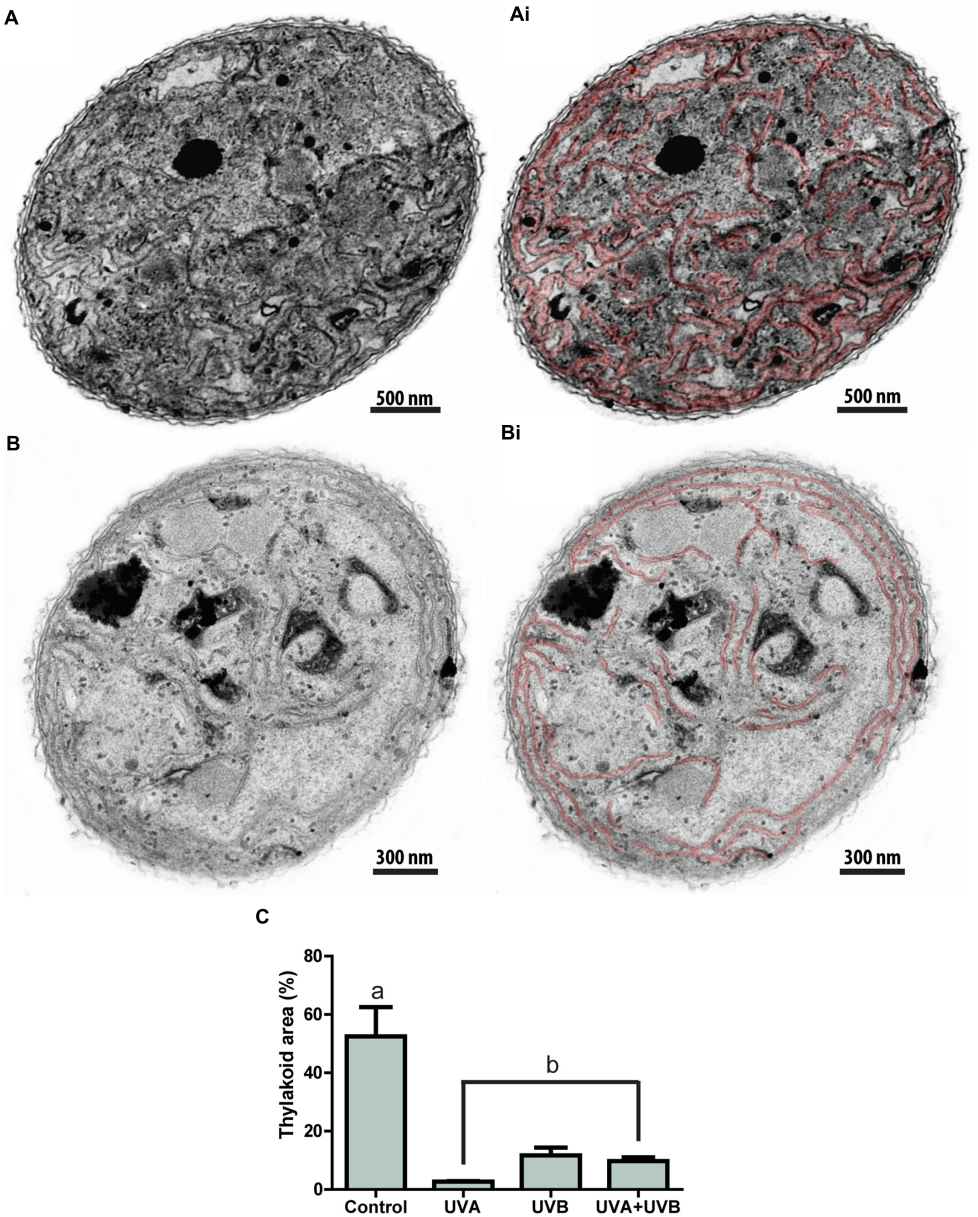
**Cyanobacterium thylakoid membranes are drastically affected by UV radiation.** While the cytoplasm of control **(A)**, untreated *Cylindrospermopsis raciborskii* shows a high amount of thylakoid membranes, these structures were greatly reduced after exposure to UV radiation **(B)**. Membranes are highlighted in red in **(Ai)** and **(Bi)**. In **(C)**, quantitative analyses show a significant reduction of thylakoid areas induced by all UV treatments. Letters indicate a significant difference (*P* < 0.05). Cells were fixed in a mixture of glutaraldehyde and paraformaldehyde and prepared for TEM. A total of 64 electron micrographs were evaluated and thylakoid areas measured using software Image J 1.41.

To investigate ultrastructural alterations underlying cell death in *C. raciborskii*, samples of cyanobacteria were prepared for TEM after 6 h of UV treatment. All three UV treatments induced marked alterations of the cyanobacteria ultrastructure when compared to the control. The most evident changes were related to photosynthetic structures. Thylakoids were drastically damaged by UV radiation, showing significant reductions in cytoplasmic area occupied by these membranes of 95.8, 77.6, and 81.3% for UVA, UVB, and UVA + UVB treatments, respectively, compared to the untreated control group (*P* < 0.05; **Figure 5**).

**FIGURE 6:**
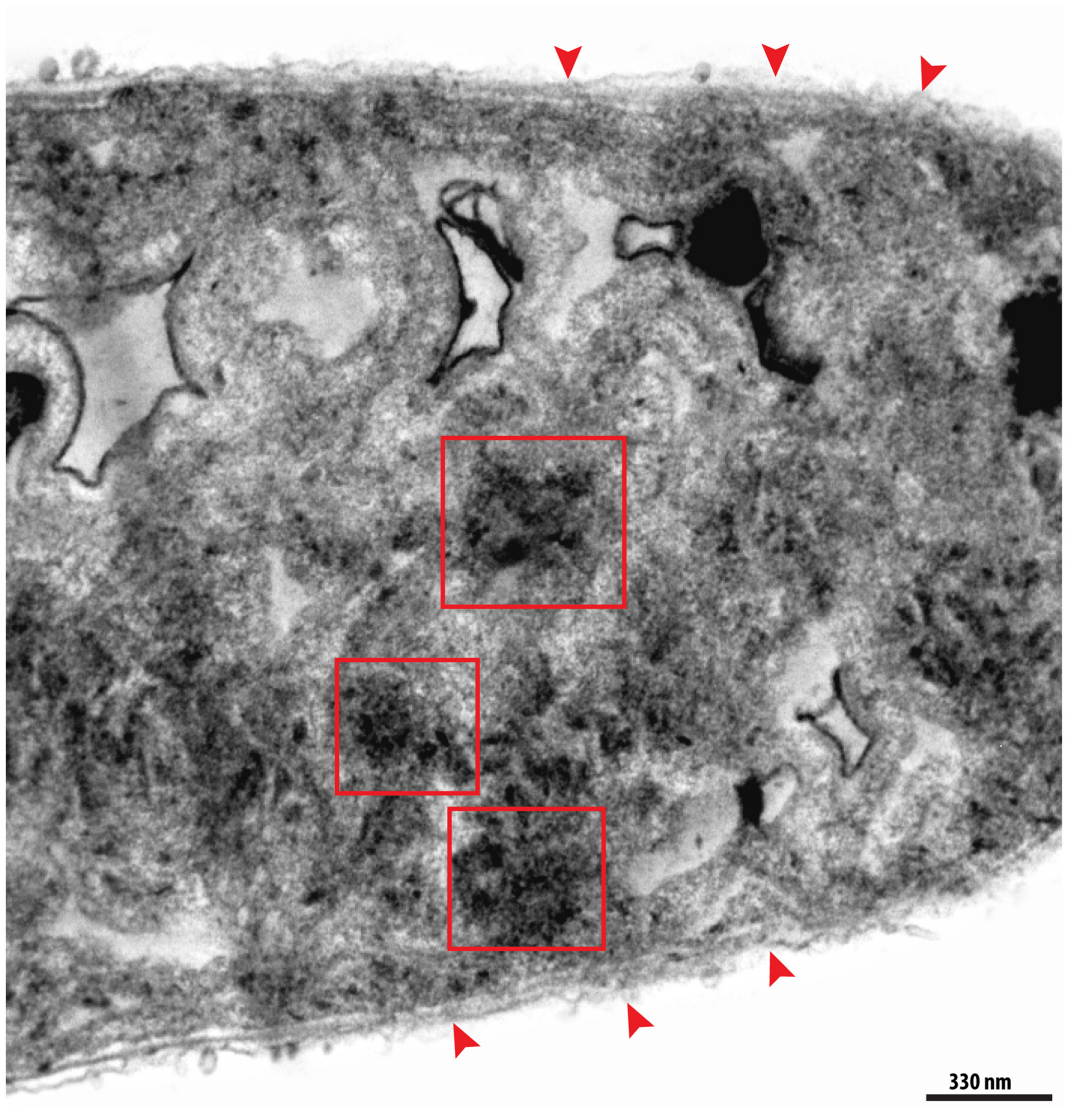
**Electron micrograph of a representative degenerating cyanobacterium.** In addition to a clear loss of thylakoid membranes, aggregates of dissolving phycobilisomes (red boxes) were observed in the cytoplasm. Note the loss of the integrity of the cell envelope (arrowheads).

Phycobilisomes and polyphosphate granules were also altered. In control cells phycobilisomes could be clearly identified as individual electron-dense dots, uniformly distributed in association with thylakoid membranes (**Figures 4A,Aii**). However, in UV-treated cells, phycobilisomes were found disarranged and frequently formed amorphous aggregates. These were dispersed in the cytoplasm, as mainly seen in the UVA-treated group (**Figure 6**).

**FIGURE 7:**
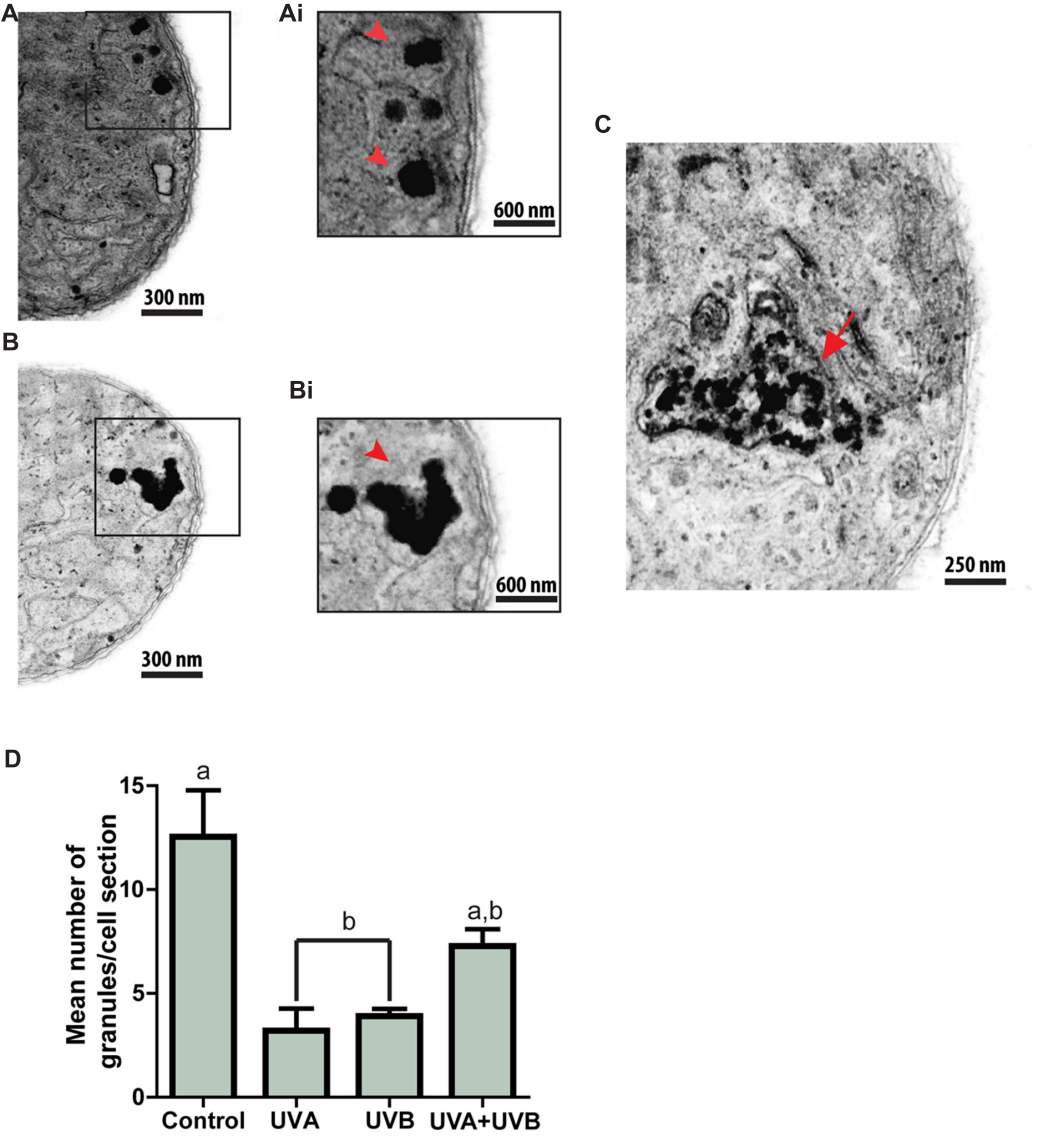
**Ultraviolet radiation induces disarrangement of *C. raciborskii* polyphosphate granules.** Polyphosphate granules (boxed area in **(A)** and **(B)** and arrowheads in **Ai** and **Bi**) are seen as intact structures in control cells **(A,Ai)**, and as disrupted structures in UV-treated cells **(B,Bi)**. A polyphosphate granule is seen at an advanced stage of disintegration [**(C)**, arrow]. A graph of the number of polyphosphate granules per cell for each group is shown in **(D)**. A total of 64 electron micrographs were evaluated and the number of granules per section quantitated. A total of 273 granules were counted. Data are expressed as mean ± SD. Letters indicate significant differences between means (*P* < 0.05).

Polyphosphate granules also appeared as disarranged structures (**Figure 7**). In control cells, such granules generally appeared having round forms (**Figures 7A,Ai**), but after radiation exposure, most had lost their morphological characteristics, and presented as amorphous structures (**Figures 7B,C**). Quantitative TEM analysis revealed that cells exposed to UVA or UVB showed a significant reduction in the number of polyphosphate granules per cell section compared to the control group (*P* = 0.0008; **Figure 7D**); this reduction corresponded to 66.87 and 63.76% for UVA- and UVB-treated groups, respectively. However, the number of granules did not significantly vary between control and UVA + UVB groups (**Figure 7D**). The total number of polyhedral bodies and gas vesicles also did not vary between groups (*P* > 0.3366 and *P* > 0.810, respectively).

## Discussion

The current study shows that UV radiation induces death of the cyanobacterium *C. raciborskii*, and that this process occurs through directly damaging cell structures. It appears that the entire photosynthetic cell apparatus of *C. raciborskii* is a target of this radiation, in conjunction with the cell envelope and structures related to cellular metabolism (polyphosphate granules).

The effect of UV radiation, especially UVB, has been evaluated in different species of cyanobacteria (reviewed in Singh et al., 2010), but not in *C. raciborskii*. For example, growth of the cyanobacteria, *Oscillatoria priestleyi* and *Phormidium murrayi*, was suppressed by 100 and 62%, respectively, following exposure to UVB radiation (Quesada and Vincent, 1997). Similarly, Han et al. (2003) reported the inhibition of growth of a rice-field cyanobacterium *Anabaena sp*. during exposure to UVA + UVB radiation, while solar UV radiation inhibited *Anabaena sp*. PCC 7120 by up to 40% (Gao et al., 2007). Other work with two cyanobacteria (*Nostoc muscorum* and *Phormidium foveolarum*) found that while a UVB dose of 1.0 μmol m^-2^ s^-1^ induced a 14–21% growth decrease, treatment using a lower UVB dose (0.1 μmol m^-2^ s^-1^) did not influence growth (Singh et al., 2012).

A study with several strains of cyanobacteria demonstrated a growth decrease of 48% after 30 min of exposure to UVB alone (14.4 ± 1 Wm^-2^), without the addition of PAR (Kumar et al., 2003); moreover, all cell strains died after 90 min of exposure (Kumar et al., 2003). Using a lower intensity of UVB (3 Wm^-2^) and another cyanobacterium strain (*Artrospira platensis)*, other groups have observed 28 and 40% growth decreases after 30 and 60 min of exposure, respectively (Ganapathy et al., 2015).

Here, we used two approaches to investigate the effect of UV radiation on *C. raciborskii* growth/death: (i) a classical cell concentration evaluation, which demonstrated a significant reduction in cell numbers in response to UVA + UVB and UVA treatments (**Figure 1**), and (ii) a cell viability test, which identified that all treatments, including UVB, were able to trigger significant cyanobacterial death (**Figure 2**). This means that while UVA and UVA + UVB led to *C. raciborskii* lysis, as indirectly shown by the cell concentration analysis, all treatments were able to elicit cellular changes indicative of cell death, i.e., loss of plasma membrane integrity. Therefore, while UVB radiation did not induce cell rupture, it still caused a loss of cell viability, although to a lesser extent compared with UVA and UVA + UVB treatments (**Figure 2B**). Thus, cell viability data were important in allowing the detection of cell death-related changes prior to cell lysis (Pearl, 2000; Agusti et al., 2006).

The use of markers for cell viability also enabled us to demonstrate the presence of viable and non-viable cells within the same cyanobacterial filament (**Figure 2C**). This likely represents the initial response of individual cells to UV radiation. It is possible that higher UV doses and/or longer times to UV exposure lead to death or inactivation of all cells within the filament.

Ultrastructural studies of cyanobacteria are still scarce, although such studies would be of great importance in better understanding the biology and diversity of these organisms, and in providing insights into their ecological responses. The ultrastructure of *C. raciborskii* was analyzed here in detail by TEM. We used a method of pre-inclusion in agar with the aims of maintaining the optimal preservation of cells and of reducing artifacts caused by mechanic damage and the loss of specimens during sample manipulation (Melo et al., 2007; Silva et al., 2014). Untreated cells exhibited subcellular details such as an elaborate three-layered envelope, and a cytoplasm containing an intricate membrane system (thylakoids), with associated phycobilisomes and other structures such as polyphosphate granules and carboxysomes (**Figures 3** and **4**). Cells were tightly arranged within each filament (**Figure 4**).

The thylakoid membranes of cyanobacteria are the major sites of respiratory electron transport, as well as photosynthetic light reactions (reviewed in Mullineaux, 2014). Knowledge of the arrangement and number of thylakoids is taxonomically important (Whitton, 1972; Lang and Whiton, 1973) because these are considered to be stable cyanobacterium features (Komárek and Vaslavská, 1991). For instance, in contrast to other cyanobacteria, such as *Annamia toxica gen. et sp. nov.*, which display concentric thylakoids (Nguyen et al., 2013), these structures are irregularly arranged in *C. raciborskii* (**Figures 3F** and **4A,Ai**).

Ultrastructural analyses identified subcellular structural targets of UV radiation. The main finding was the detection of accentuated damage to thylakoid membranes (**Figure 5**). These data are in agreement with previous studies that showed that treatment with UVB for 2 h affected thylakoid membranes in cyanobacteria of the genus *Synechococcus sp*. (Chauhan et al., 1998). Structural alterations and subsequent reductions of these specialized structures were also observed in eukaryotic phytoplankton affected by UVB (Meindl and Lütz, 1996; Lütz et al., 1997).

In parallel to the thylakoid loss, we observed structural disarrangement of photosynthetic pigments (phycobilisomes; **Figure 6**). A study using molecular markers also reported the dissociation of phycobilisomes from thylakoid membranes when *Synechococcus sp.* were exposed to UVA + UVB + PAR (UVA: 4.3 Wm^-2^ and UVB 0.86 Wm^-2^) (Six et al., 2007). Previous studies using only UV radiation (without PAR addition) suggested that phycobilisomes and photopigments on thylakoid membranes served as specific targets for UV radiation, particularly UVB (Rajagopal et al., 1998; Gupta et al., 2008; Ganapathy et al., 2015). It is believed that UV radiation affects the photosynthetic apparatus in some way (directly or indirectly through reactive oxygen species [ROS]) by, for instance, acting on proteins of thylakoid membranes, or on photosynthetic pigments, and/or on proteins that link these pigments to thylakoid membranes. For example, under UVB stress, the cyanobacterium, *Spirulina platensis*, showed both structural (membrane distortions) and molecular (pigment protein complexes) alterations in thylakoid membranes (Rajagopal et al., 1998; Gupta et al., 2008). In *Arthrospira platensis*, levels of photosynthetic pigments, total chlorophyll, total carotenoids and *c*-phycocyanin decreased after long exposure times (9 h) to 3.0 Wm^-2^ UVB radiation (Ganapathy et al., 2015).

UVB radiation is the most energetic region of the UV spectrum that reaches ground level, affecting mainly cyanobacterial DNA (Kumar et al., 2004). In this regard, we therefore expected greater damaging effects with such treatment. However, our results demonstrated that UVB exposure was less deleterious in comparison to UVA and UVA + UVB treatments. This fact might be due to the UVB intensity used. While most studies used higher UVB doses compared to those of our study (Quesada and Vincent, 1997; Sinha et al., 1997; Kumar et al., 2003; Fijalkowska and Surosz, 2005), the intensities of UV radiation used in the current experiments corresponded well with UVA and UVB intensities on a sunny day in the Brazilian southeast region during autumn (UVA: 11.8 W m^-2^ and UVB: 0.54 W m^-2^). Therefore, the UV intensities adopted here can be considered moderate intensities when compared to those of summer in regions of high and middle latitudes (UVA: 45–50 W m^-2^ and UVB: 7–8 W m^-2^) (Castenholz and Garcia-Pinchel, 2000). We speculate that UVB at the intensity used in the present study may have had a potential photoprotective effect (Xu and Gao, 2010). Indeed, mechanisms of protection are triggered by light since high light intensities are usually accompanied by high intensities of biologically active UVB radiation in natural environments (Ding et al., 2013). However, cyanobacteria contain amino acids that provide UV protection (Sinha and Häder, 2008), and under only UV radiation are able to produce antioxidant enzymes (Ganapathy et al., 2015) and increase membrane lipid unsaturation (Gupta et al., 2008). These have been suggested to be protective mechanisms that keep cells alive under stress.

On the other hand, it is known that UVB effects are dependent on the wavelength incidence into this spectrum (Mitchell and Karentz, 1993). In fact, Meindl and Lütz (1996) observed that damage to the morphology and development of *Micrasterias denticulata* (Chlorophyceae) by UVB occurred within a short time frame when cells were treated with a wavelength shorter than 275 nm. Such results may explain the lower effect UVB had compared to UVA in our experiments, since the lamp used had an emission peak at a wavelength of 312 nm, which corresponds to an area of greater length and less energy in the UVB radiation spectrum. Other possibilities to be considered are differences in the sensitivity of cyanobacteria to various UV wavelength bands. Dissimilarities in UVB responses suggest a species-specific effect, which can be an important driver of community structure and the succession of species (Wangberg et al., 1996; Quesada and Vincent, 1997). Moreover, it has been demonstrated that the production of specific toxins by cyanobacteria may prevent cellular damage under moderate UV intensities. For example, a toxic strain of cyanobacteria *Microcystis aeruginosa* (a microcystin producer) increases bacterial fitness in high light intensities and shows a protective effect against UVB radiation (Zilliges et al., 2011; Ding et al., 2013).

In our experiments, UVA induced the most drastic alterations in *C. raciborskii* ultrastructure, both in thylakoid membranes and in photosynthetic pigments. In general, exposure to UVA was reported to cause the photo-inhibition of a natural population of cyanobacteria (Kim and Watanabe, 1993). However, the effects of UVA on other aspects of cyanobacteria, including ultrastructure, are poorly understood and their significance awaits further investigation. Using TEM, we also found UV radiation affected other structures related to cellular metabolism, such as polyphosphate granules. The changes noted in these granules were another structural indication that the cell was in the process of dying in response to UV radiation. These cytoplasmic granules exhibited disarrangement, dissolution and a decrease in their number, mainly in response to UVA and UVB treatments (**Figure 7**). Polyphosphate granules have important functions in phosphate storage and energy metabolism, since phosphate is involved in the biosynthesis of nucleic acids, phospholipids and ATP (reviewed in Allen, 1984; Achbergerova and Nahalka, 2011). Moreover, *C. raciborskii* demonstrates a large capacity to stock phosphorous (as phosphate), which gives a competitive advantage to these cyanobacteria (Padisák, 1997). In accordance with the viability results, we also identified UV-induced ultrastructural changes of the cyanobacterial cell envelope (**Figure 6**), likely due to the absorption of UV radiation by membrane proteins, eventually resulting in cell death (Sinha et al., 1996).

Altogether, we demonstrated clear alterations in the viability and photosynthetic structures of *C. raciborskii* induced by UV radiation fractions. This study facilitates our understanding of the subcellular organization of this interesting and ecologically important cyanobacterium species, and identifies specific intracellular targets of UVA and UVB radiation. Moreover, our study opens new perspectives to investigate at high resolution possible signals of cell damage induced by UV radiation in *in situ* organisms based on TEM and also to evaluate the critical levels of UV to cell physiology and morphology that may leads to cell death in natural ecosystems.

In summary, our results reinforce and expand the importance of UV radiation as an environmental stressor and recognize subcellular structures involved in UV radiation-induced-cell damage, such as thylakoid membranes, phycobilisomes and polyphosphate granules. The knowledge of physiological responses at cellular level in cyanobacterial populations affected by UV radiation is important to understand the connection of environmental changes and persistence of species in aquatic ecosystems. Future investigations may link the individual physiology and the environmental conditions.

## Conflict of Interest Statement

The authors declare that the research was conducted in the absence of any commercial or financial relationships that could be construed as a potential conflict of interest.
