# Supplementary material for: Potential effects of UV radiation on photosynthetic structures of the bloom-forming cyanobacterium Cylindrospermopsis raciborskii CYRF-01
Source: Front Microbiol. 2015 Oct 30;6:1202. doi: 10.3389/fmicb.2015.01202 (PMC4627488; doi:10.3389/fmicb.2015.01202)
Supplement: Supplementary file 1 [file Image_1.PDF]

Supplementary Material

Potential effects of the UV radiation on the photosynthetic structure of the bloom-forming cyanobacterium *Cylindrospermosis raciborskii* CYRF-01.

Natália P. Noyma[1#], Thiago P. Silva[2#], Hélio Chiarini-Garcia[3], André M. Amado[4], Fábio Roland[1] and Rossana C. N. Melo[2*]

[1]Laboratory of Aquatic Ecology, Department of Biology, Federal University of Juiz de Fora, Juiz de Fora, MG, Brazil
[2]Laboratory of Cellular Biology, Department of Biology, Federal University of Juiz de Fora, Juiz de Fora, MG, Brazil
[3]Laboratory of Structural Biology and Reproduction, Department of Morphology, Federal University of Minas Gerais, Belo Horizonte, MG, Brazil
[4]Laboratory of Limnology, Department of Oceanography and Limnology, Federal University of Rio Grande do Norte, Natal, RN, Brazil

*Correspondence: Dr. Rossana C. N. Melo, Laboratory of Cellular Biology, Department of Biology, Federal University of Juiz de Fora, Rua José Lourenço kelmer, Juiz de Fora, 36036-900, Brazil
rossana.melo@ufjf.edu.br

# These authors contributed equally to this work.

1. Supplementary Figure

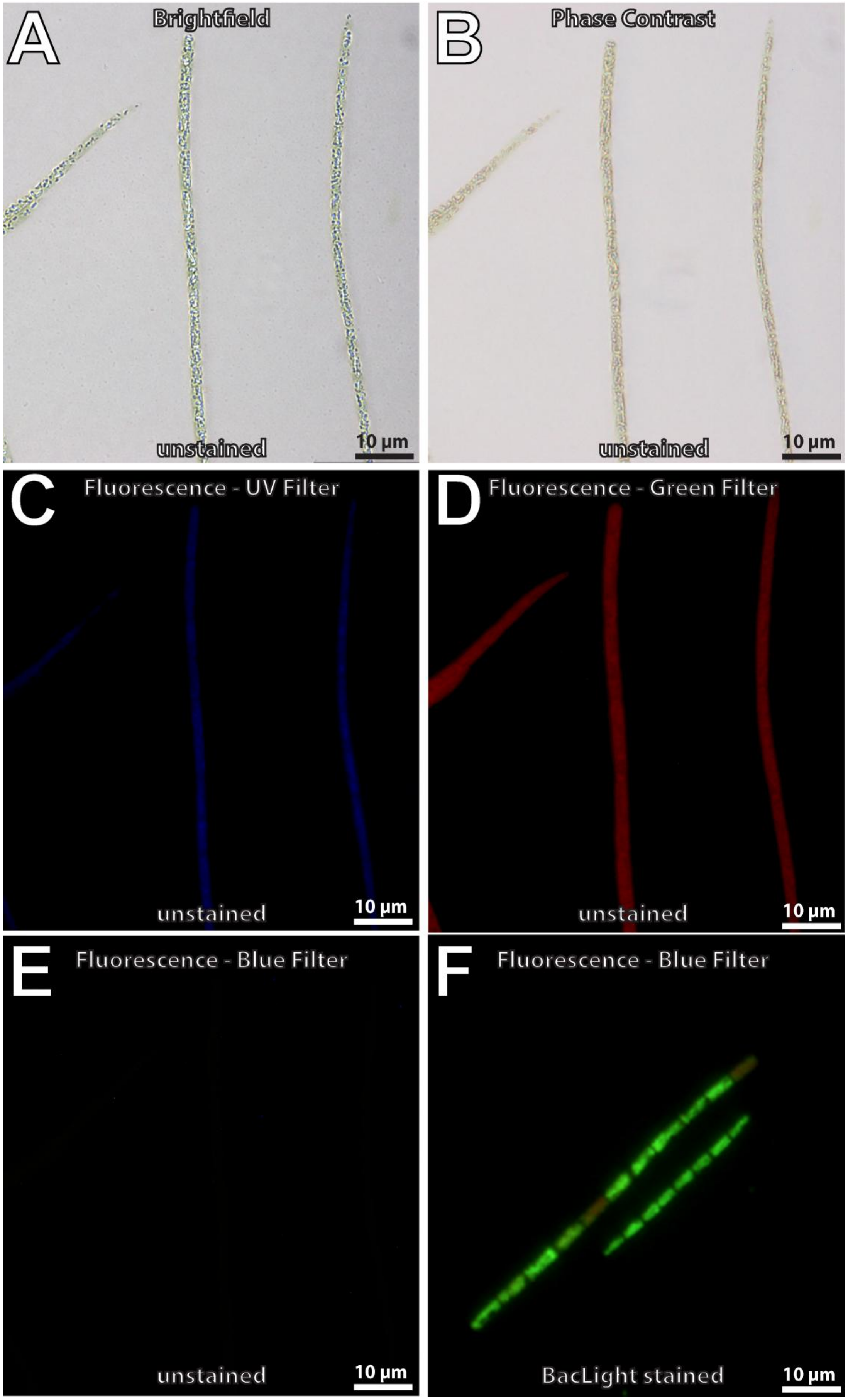
A
Brightfield
unstained
10 µm
B
Phase Contrast
unstained
10 µm
C
Fluorescence - UV Filter
unstained
10 µm
D
Fluorescence - Green Filter
unstained
10 µm
E
Fluorescence - Blue Filter
unstained
10 µm
F
Fluorescence - Blue Filter
BacLight stained
10 µm

Supplementary Figure 1. *Cylindrospermopsis raciborskii* visualized under bright-field (A), phase contrast (B) and fluorescence (C-F) microscopy. Samples were collected from non-treated cultures and slides were prepared by cytocentrifugation. (A-E) show identical fields of unstained cyanobacteria. Note that *C. raciborskii* exhibits autofluorescence with the ultraviolet (C) and green (D) filters, but not with the blue filter (E). In (F), cyanobacteria are seen under blue filter after staining with *BacLight* dyes (Syto 9 + propidium iodide). Observe that most filaments exhibit green fluorescence, indicative of live cells.
